# Research on genetic diversity and genomes of Miscanthus for optimizing their biotechnological potential

**DOI:** 10.18699/vjgb-26-20

**Published:** 2026-07

**Authors:** I.V. Chadaeva, D.I. Karetnikov, A.Yu. Pronozin, E.S. Starodubtseva, N.A. Omeljanchuk, A.V. Kochetov, D.A. Afonnikov

**Affiliations:** Institute of Cytology and Genetics of the Siberian Branch of the Russian Academy of Sciences, Novosibirsk, Russia; Institute of Cytology and Genetics of the Siberian Branch of the Russian Academy of Sciences, Novosibirsk, Russia; Institute of Cytology and Genetics of the Siberian Branch of the Russian Academy of Sciences, Novosibirsk, Russia; Institute of Cytology and Genetics of the Siberian Branch of the Russian Academy of Sciences, Novosibirsk, Russia; Institute of Cytology and Genetics of the Siberian Branch of the Russian Academy of Sciences, Novosibirsk, Russia; Institute of Cytology and Genetics of the Siberian Branch of the Russian Academy of Sciences, Novosibirsk, Russia; Institute of Cytology and Genetics of the Siberian Branch of the Russian Academy of Sciences, Novosibirsk, Russia

**Keywords:** Miscanthus, new agricultural crop breeding, RNAseq sequencing, transcriptome, genetic diversity, stress tolerance, markers, databases, biofuels, biotechnology and industry, мискантус (Miscanthus), селекция новой сельскохозяйственной культуры, секвенирование RNAseq, транскриптомы, генетическое разнообразие, устойчивость к факторам стресса, маркеры, базы данных, биотопливо, биотехнологии и промышленность

## Abstract

Plants of the genus Miscanthus are a promising perennial energy crop, combining high biomass productivity, resistance to abiotic stress, and low agricultural technology requirements. This review summarizes recent advances in genomic and transcriptomic studies of the molecular genetic mechanisms underlying the economically valuable traits of Miscanthus. Data on whole-genome assemblies of key species – M. sinensis (Msi), M. sacchariflorus (Msa), M. floridulus (Mfl), and M. lutarioriparius (Mlu) – are presented using various technologies (Illumina, PacBio, Oxford Nanopore, Hi-C). Genomic studies have revealed the complex evolution of the genus, including paleoallopolyploidy, chromosomal fusions, and duplications, which accounts for the high genetic diversity of these species. Their genome assemblies at the complete chromosome level have become the basis for comparative genomics, establishing taxonomic relationships (including the recognition of Mlu as a subspecies of Msa and Mfl as a subtype of Msi), and studying synteny with related crops such as sorghum. The information on the Miscanthus genome allows for the complete and accurate identification of a set of genes targeting breeding for the most important biotechnological traits. At the same time, the commercial hybrid M. × giganteus (M × g) is characterized by extremely low levels of genetic polymorphism, making it vulnerable to pathogens and climate fluctuations. The integration of genetic polymorphism data, phylogeography, and functional annotation of genomes opens up opportunities for the development of new, productive, and environmentally friendly Miscanthus varieties through interspecific crossings, ploidy modification, and genetic engineering. These advances contribute to the optimization of the biotechnological potential of Miscanthus for the production of biofuels and other biomaterials and the restoration of degraded lands.

## Abstract (ru)

Представленный обзор посвящен интеграции геномных подходов в изучение растений рода Miscanthus. Мискантусы представляют собой многолетние травы, перспективные для создания энергетических культур, характеризующихся высокой продуктивностью биомассы, устойчивостью к абиотическим стрессам и низкими требованиями к агротехнологиям. Рассматриваются методы анализа генетического разнообразия, полногеномные сборки, а также современные достижения в расшифровке молекулярно-генетических механизмов, определяющих продуктивность и стрессоустойчивость, с целью применения их в селекции. Приведены данные по полногеномным сборкам ключевых видов – M. sinensis (Msi), M. sacchariflorus (Msa), M. floridulus (Mfl) и M. lutarioriparius (Mlu) – с использованием различных технологий (Illumina, PacBio, Oxford Nanopore, Hi-C). Геномные исследования выявили сложную эволюцию рода, включающую палеоаллополиплоидию, хромосомные слияния и дупликации, что обусловливает высокое генетическое разнообразие у этих видов. Полные хромосомные сборки геномов их стали основой для сравнительной геномики, установления таксономических отношений (включая признание Mlu подвидом Msa, а Mfl – подтипом Msi) и изучения синтении с родственными культурами, такими как сорго. Полученная информация о геноме мискантусов позволяет с высокой полнотой и точностью идентифицировать набор генов, целевых для селекции по наиболее важным биотехнологическим признакам. В то же время коммерческий гибрид M. × giganteus (M × g) характеризуется крайне низким уровнем генетического полиморфизма, что делает его уязвимым к патогенам и климатическим колебаниям. Совокупность данных о генетическом полиморфизме, филогеографии и функциональной аннотации геномов открывает возможности для создания новых, продуктивных и экологически безопасных сортов мискантуса через межвидовые скрещивания, модификацию плоидности и генную инженерию. Эти достижения способствуют оптимизации биотехнологического потенциала мискантусов в производстве биотоплива и других биоматериалов, а также восстановлению деградированных земель.

## Introduction

The genus Miscanthus (silvergrass) comprises tall perennial grasses with short or long rhizomes and a rigid rachis and belong to the family Poaceae, specifically to the tribe Andropogoneae. Miscanthus species, are native to the temperate monsoon climates of East Asia (the Russian Far East) (Dorogina et al., 2025), as well as subtropical and tropical regions of East Asia, Southeast Asia, and the Pacific Islands, with several species extending to tropical Africa (Brosse et al., 2012; Wang et al., 2021). The highest species diversification is found in East Asia, particularly in China and Japan (Hodkinson et al., 2015).

Members of the genus Miscanthus exhibit high genetic diversity (Clark et al., 2019) and heterogeneity in genome structural organization, with ploidy levels ranging from 2 to 6 and variable chromosome numbers (for example, in the species reviewed here – M. sacchariflorus (Msa), M. lutarioriparius (Mlu), M. floridulus (Mfl), and M. sinensis (Msi) – the chromosome number is 19, whereas in Asian species such as M. fuscus, M. nepalensis, and M. nudipes, and African species such as M. ecklonii, M. junceus, M. sorghum, and M. violaceus, chromosome numbers may be 10 or 15) (Hodkinson et al., 2015). Msi is a genetic diploid (2n = 2x = 38) with a genome size of 1C = 2.4–2.6 Gb (Rayburn et al., 2009); the closely related Msa occurs in both diploid (2n = 2x = 38) and tetraploid (2n = 4x = 76) forms (Mitros et al., 2020). The high genetic diversity within the genus Miscanthus is further enhanced by their ability to readily form hybrids, even between species of different ploidy levels (Clark et al., 2015, 2019).

Miscanthus species are among the most promising crops for biofuel production, owing to several key traits relevant to industrial applications: high photosynthetic efficiency, effective nutrient and water use, high biomass yield (Wang et al., 2021), and broad adaptability to diverse climatic conditions and soil types (Clifton‐Brown et al., 2017). These characteristics enable the use of Miscanthus as an important industrial crop across various business activities, including construction, manufacturing, and agriculture (Mironova et al., 2023) (see the Figure). For bioenergy and biomass feedstock purposes, the most important species are Msa and Msi, as well as their interspecific hybrid M. × giganteus (M × g), classified as a nothospecies (Trieu et al., 2022).

**Fig. 1.**
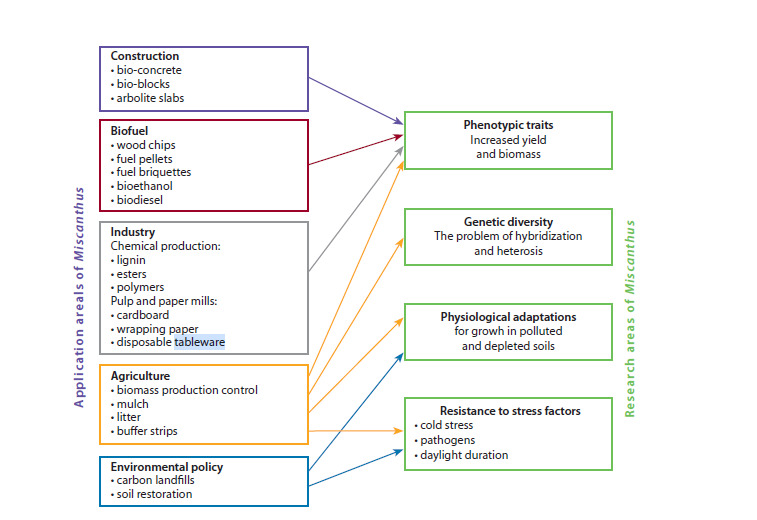
Miscanthus in human economic activities and key research areas for harnessing its genetic potential as a novel biotechnological crop.

In construction, Miscanthus serves as a source of biofiber which, when added to concrete, improves its mechanical properties as well as its sound and thermally insulating performance (Chen Y.X., Yu, 2024). The biomass yield of Miscanthus is comparable to that of maize (Sanford et al., 2016), higher than that of several other crops – including switchgrass (Panicum virgatum), poplar (Populus sp.), and willow (Salix sp.) – and exceeded only by eucalyptus (Eucalyptus sp.) (Li W. et al., 2018).

Miscanthus biomass comprises cellulose, hemicellulose, lignin, and various associated compounds (Schäfer et al., 2019). This composition underpins the active use of Miscanthus as a raw material source for the pulp and paper industry and for the production of eco-friendly packaging (Cappelletto et al., 2000). Furthermore, Miscanthus biomass serves as a feedstock for producing a wide range of reagents and polymers, including cellulose derivatives, ethanol, 5-hydroxymethylfurfural, furfural, and various phenolic compounds (Shavyrkina et al., 2023) (see the Figure).

In agriculture, Miscanthus is used as a source of biochar to improve soil condition and enhance fertility (Nagel et al., 2019). Miscanthus straw treated with Trichoderma can serve as a peat substitute for strawberry cultivation (Debode et al., 2018). It is also employed as a substrate for the anaerobic digestion of cattle and swine manure in biogas fermenters (Jury et al., 2022) and as bedding for cattle, which reduces bedding costs (Van Weyenberg et al., 2016).

One of the most important characteristics of Miscanthus is its low requirement for cultivation conditions. This enables the use of this crop for soil remediation (Grzegórska et al., 2023), particularly in areas contaminated with pollutants such as organochlorine pesticides (Mamirova et al., 2021), heavy metal salts (Andrić et al., 2025), and polycyclic aromatic hydrocarbons (Técher et al., 2012). The ability of Miscanthus to accumulate toxins in its roots is currently under close scientific scrutiny and is being actively applied in practice (Nsanganwimana et al., 2021).

The primary objectives in Miscanthus breeding can be summarized as follows: 1) overcoming the low genetic diversity of commercial hybrids (such as M × g); 2) preventing invasiveness through reproductive isolation (sterility) and specific containment measures for vegetatively propagated, long-rhizome Miscanthus species, such as Msa; 3) developing new hybrids with novel beneficial traits through interspecific crosses (e. g., Msi × Msa, Msi × Mfl); and 4) leveraging modern biotechnologies, including genetic modification and in vitro regeneration.

The efficiency of classical breeding methods can be substantially enhanced through the application of modern genetic approaches and omics technologies (Yang et al., 2021). In this review, we examine the use of genomic technologies to study genetic diversity in Miscanthus, reconstruct genomes, and identify target genes for plant breeding programs.

## Studying genetic diversity in Miscanthus

Studying diversity primarily helps to link genetic relatedness between various taxa of this genus with their geographical distribution. The main goal of such studies is to identify patterns of distribution of various Miscanthus species, identify centers of origin and genetic diversity, and determine the influence of climatic factors on evolutionary mechanisms of speciation. One of the main questions is also establishing the relationships between species of the genus Miscanthus, since despite intensive research, their taxonomic position and the evolution of species Msi, Mfl, Msa, and Mlu remain not fully understood (Sun et al., 2010; Sang, Zhu, 2011; Hodkinson et al., 2015).

In the study (Cichorz et al., 2014), an analysis of genetic diversity of samples of four Miscanthus species from Poland was conducted: M × g, Msi, Msa, and Mfl. ISSR (inter-simple sequence repeats) and RAPD (random amplified polymorphic DNA) markers were used for the analysis. For M × g, low genetic diversity was shown, consistent with its origin from a single clone. In contrast, Msi is characterized by high genetic diversity, which is confirmed in the study of Msi populations from Southwest China using SRAP (sequence-related amplified polymorphism) markers (Nie et al., 2014).

A large-scale study of Msi populations in China based on the analysis of 459 samples from various geographical regions of the country using SSR markers revealed high genetic diversity, indicating the great potential of Msi as a genetic resource for breeding (Zhao et al., 2013). Interestingly, Msi chloroplast DNA demonstrates comparatively low genetic variability, which is explained by greater susceptibility to random drift and a smaller effective population size for chloroplast DNA compared to nuclear DNA (Yan et al., 2015).

In the study (Li S.-S. et al., 2019), 100 populations of four Miscanthus species, Msi, Mlu, Mfl, and Msa, growing in China from northern to southern regions were investigated. Two genetically homogeneous groups were identified. The first included plants of species Msa and Mlu, the second – Mfl and Msi. Based on genetic diversity data, possible routes of Miscanthus population evolution were modeled. Results showed that species from the first group underwent a range contraction during the Last Glacial Maximum (26–19 thousand years ago) and then its gradual expansion, whereas species of the second group have gradual range expansion from the interglacial period (129–116 thousand years ago) to the present, expanding to the southern China regions. The authors of this study proposed a hypothesis on the origin of Mfl, according to which Mfl species originated from Msi ancestors in southeastern China (Li S.-S. et al., 2019). This is confirmed by the shared geographical distribution of these species, as well as their positions on the phylogenetic tree, where the Mfl population cluster is located within the Msi cluster

A large-scale research was dedicated to investigating the Msa and Mlu population, which included 764 samples from Russia, China, South Korea, and Japan (Clark et al., 2019). The aim of the work was to study the diversity, origin, and evolution of representatives of these Miscanthus species. DNA analysis was based on RAD-seq (restriction site-associated DNA sequencing) technology, and additionally, ploidy analysis was performed using flow cytometry. As a result, six genetic groups were identified: three groups were represented by diploid varieties growing predominantly in Northeast China, Korea, and Russia; the remaining three groups turned out to be tetraploids growing in Northern China, Korea, and Japan.

According to the researchers, the modern Msa population formed during the last glacial period from an ancestral population, whose habitat was located in Eastern China, in an area now covered by the Yellow and East China Seas. Also, this work showed that polyploidization events occurred independently for the Japanese and mainland Msa groups. Analysis of plastid haplotypes revealed 56 unique variants between Msa and Msi. Nine of them are intermediate between the most common haplotypes of the compared species (Clark et al., 2019).

The results of this work also confirmed the assumption that Mlu is a subspecies of Msa, originating in the Yangtze River basin area, and not a separate Miscanthus species. The study (Clark et al., 2019) demonstrated higher genetic diversity of the species Msa compared to Msi, indicating a difference in the population history of these two species.

In the study (Chen et al., 2022), using SLAF-seq (specificlocus amplified fragment sequencing) technology, genetic differences between plants belonging to species Msi, Mlu, and Msa were investigated. Analyzing 50 samples of each of these three species, the authors showed that the genetic distance between Mlu and Msi turned out to be smaller than the genetic differences within the Mlu population itself. Their results are consistent with the results of the study L.V. Clarket al. (2019).

A large-scale population analysis of various Miscanthus samples, including about 2,800 samples from China, Korea, Russia, and Japan, was conducted using GBS (genotypingby- sequencing) in the study T. Mitros et al. (2020) along with the assembly of the Msi genome. Results showed high genetic diversity among representatives of this genus. The analysis also allowed clarifying taxonomic relationships within it. According to this work, the populations of species Msi split into two large clusters, one of which includes representatives growing in Japan, and the other – in China and Korea.

Plants of species Msa represent a separate distinct group, which demonstrate less diversity. Representatives of the M × g population occupied an intermediate position between Msi and Msa, combining genetic components of both species. The genetic diversity of Msi significantly exceeds that of a number of other species, M. transmorrisonensis and Mfl. This allowed the authors to conclude that the latter two taxa can be considered as subtypes of Msi, belonging to the subpopulation from Japan (Mitros et al., 2020).

In the study (Zhang et al., 2021), aimed at sequencing and analyzing the Mfl genome, genetic diversity was evaluated for 75 accessions of Mfl, M × g, Msa, Mlu, Msi and a number of hybrid plants obtained by crossing these species. Analysis of these populations showed that Mfl and Msi represent separate groups genetically distant from other representatives of the genus, and plants Msa and Mlu form a separate cluster, confirming the status of Mlu as a subspecies of Msa.

Thus, analysis of genetic diversity based on the use of whole-genome sequencing of DNA fragments allowed establishing the population history of Miscanthus species and clarifying taxonomic relationships within representatives of this genus: classifying the taxon Mlu as a subspecies of Msa, and taxa M. transmorrisonensis and Mfl as subtypes of Msi. The wide genetic variability of Miscanthus genus revealed makes it possible to conduct targeted breeding for agricultural and industrial purposes (Hodkinson et al., 2015; Trieu et al., 2022).

Genetic and geographical data serve as a basis for creating breeding programs aimed at developing varieties adapted to different climatic zones. In a recent study (Dorogina et al., 2025), an investigation was conducted on the possibility of cultivating Msi samples from monsoon climate regions under condition of the forest-steppe zone in Western Siberia. During the trials, two samples, S1 and S2, were selected, which formed viable seeds during the short growing season characteristic of the Siberian continental climate, from which two generations, G1 and G2, were obtained. These samples also had accelerated rates of seasonal development and a more compact habitus. They formed less vegetative mass and began to form generative organs earlier than typical representatives of Msi.

Study of genetic variability in generation G1 showed complete uniformity of genotypes. In generation G2, on the contrary, diversity was observed with the identification of five different genotype variants. As the authors state, the phenotypic and genetic variability they discovered in Msi will allow selecting forms with various economically valuable traits for further genetic improvement and development of a variety with desired traits.

## Genomic studies of genus Miscanthus

To date, several whole-genome assemblies have been generated for four Miscanthus species: Msa (De Vega et al., 2021), Mlu (Miao et al., 2021), Mfl (Zhang et al., 2021), and Msi (Mitros et al., 2020), differing in quality and completeness (see the Table).

**Table 1.**
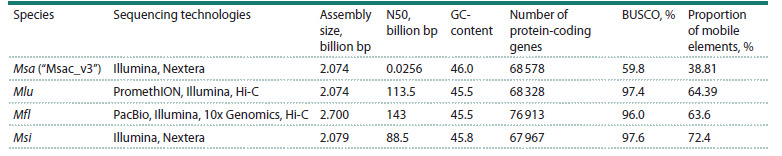
Comparative characteristics of genome assemblies in four Miscanthus species

The Table shows the general assembly parameters: sequencing technologies used, assembly size, N50 metric, guanine and cytosine content (GC content), genome and transcriptome quality assessment using BUSCO (Benchmarking Universal Single-Copy Orthologs) (Simão et al., 2015), as well as the proportion of mobile elements in the genomic sequence. Assemblies were performed using different sequencing technologies and assembly methods, which affects their accuracy and suitability for downstream analysis. Below, we present the results of genomic studies for these four Miscanthus species

**The Msa genome**

Samples from the diploid cultivar Robustus 297 were used for sequencing of the Msa genome (De Vega et al., 2021). Short-read libraries with small inserts generated using Illumina HiSeq 2500 platform were employed (~5.86 billion reads, genome coverage ~50×). For scaffold assembly, a paired-end read library with a read length of 150 bp and a large insert size (7 kb) was used, totaling 141 million read pairs.

The primary contig assembly (Msac_v2) was performed using the ABySS software (Simpson et al., 2009), yielding 17 million contigs with a total length of 3.27 Gb. As a result of scaffolding, ~589 thousand sequences were obtained with a total length of 2.54 Gb and an N50 metric of approximately 10.2 kb (see the Table). To improve assembly quality, sequences shorter than 2 kb were filtered out, and further scaffolding was performed for the remaining longer scaffolds using paired libraries from Msi data from the study (Mitros et al., 2020). Ultimately, the “Msac_v3” assembly was obtained with a length of 2.074 Gb (see the Table), consisting of 137,916 scaffolds, with the N50 increasing to 25.6 kb.

The final assembly (Msac_v3) was achieved by ordering scaffolds into chromosomes based on alignment with Msi chromosomes. Genome annotation was performed using the AUGUSTUS software (Stanke et al., 2004). The final annotation covers approximately 81 thousand genes for the Msac_v2 assembly and approximately 68 thousand for the Msac_v3 assembly. The proportion of mobile elements for the obtained assembly was 38.81 % (see the Table). For the Msa genome assembly Msac_v3, completeness based on BUSCO analysis was 55.5–59.8 % (De Vega et al., 2021).

**The Mlu genome**

The Mlu genome was assembled using multiple sequencing and assembly technologies, which ensured high-quality representation at the chromosomal level (Miao et al., 2021). The Oxford Nanopore PromethION platform was applied for sequencing yielding long DNA reads with a total size of 307.71 Gb and N50 parameter of 32.21 kb. Additionally, three Illumina libraries with different insert sizes were used, providing reads totaling 205.74 Gb, of which 172.52 Gb remained after quality filtering. Hi-C (High-throughput chromosome conformation capture) technology was implemented to order the assembled genomic sequence fragments into chromosomes.

The primary genome assembly was performed using SMARTdenovo software (Liu et al., 2021) and based solely on long Nanopore reads. The size of the primary assembly was 2.25 Gb with an N50 parameter of 1.71 Mb. At the final assembly stage, reads from all libraries and Hi-C data were incorporated, resulting in 919 scaffolds with a total size of approximately 2.074 Gb (see the Table), of which 94.3 % (approximately 1.96 Gb) were placed and oriented on 19 pseudochromosomes ranging in size from 61.78 to 150.81 Mb.

Comparison of contig sequences with BAC (bacterial artificial chromosome) sequences obtained by Sanger sequencing showed very high similarity at the 99 % level. Alignment of Illumina reads to the assembled genome indicated its high completeness and accuracy (99.8 %). The GC content of the genome was approximately 45.5 %, and mobile elements (MEs) accounted for 64.4 % of the genome (Miao et al., 2021). The total number of predicted genes was 68,328. BUSCO analysis of protein-coding gene content demonstrated a completeness of 97.4 % (see the Table). The authors performed a synteny analysis of the obtained Mlu assembly with the sorghum genome, which allowed the identification of conserved genomic regions.

**The Mfl genome**

For the Mfl genome assembly, sequencing was performed using Illumina, PacBio, and 10x Genomics platforms, and a Hi-C library was used for scaffold ordering (Zhang et al., 2021). The final assembly comprises 19 pseudochromosomes covering approximately 2.44 Gb (91 % of the genome), with a total genome size of 2.700 Gb and an N50 of 143 Mb (see the Table). The quality of the final assembly was confirmed by high coverage with short reads and high BUSCO completeness scores for protein-coding genes (96 %).

The Mfl genome is characterized by a high proportion of mobile elements (63.6 %, predominantly LTR retrotransposons). Genome analysis identified 76,913 genes, of which 71,637 (91.1 %) were functionally annotated. Comparison of Mfl with other crops, Zea mays, Oryza sativa, Sorghum bicolor, and S. spontaneum, revealed that a set of more than 13 thousand gene families was common to these cereal genomes. In turn, 2,219 gene families were unique to Mfl.

**The Msi genome**

The Msi genome was assembled to the chromosome level (Mitros et al., 2020). The total size of the diploid Msi genome is approximately 2.4–2.6 Gb. The Msi genome assembly was performed using an integrated approach involving multiple sequencing methods. The starting material was a dihaploid (DH1) Msi line, which is homozygous for genetic markers. Sequencing was performed using Illumina HiSeq 2000 technology, as well as Nextera long-insert paired-end reads and fosmid libraries on the Illumina MiSeq platform. To improve the genome assembly, read libraries obtained using Hi-C technology were employed, enabling the establishment of threedimensional contacts between chromosome fragments within the nucleus. Ultimately, a sequence of 2.079 Gb was obtained for the nuclear genome, with an assembly N50 parameter of 88.5 Mb (see the Table). In addition to the nuclear genome, the assembly of chloroplast and mitochondrial genomes was performed within this study.

For protein-coding gene prediction, bioinformatic ab initio methods and RNA-seq transcriptome library data were used. In this study, transcriptome data included 57 growth time points for three Msi DH1 tissues; additionally, transcriptome data for the M × g hybrid were used. In total, this allowed the prediction of 67,967 protein-coding genes (94.3 % localized on chromosomes), of which more than 50,000 were confirmed by RNA-sequencing data. The proportion of repetitive DNA for the obtained assembly was 72.4 % (see the Table). Assessment of annotation completeness using the BUSCO method (Simão et al., 2015) showed that 97.6 % of Embryophyta genes are represented completely in the assembly (see the Table), another 1 % are fragmented, and 64.3 % of genes are duplicated, which is consistent with the paleotetraploid nature of representatives of the genus Miscanthus (Mitros et al., 2020).

The available genome assemblies of Miscanthus species have enabled in-depth studies of the evolution of their genome structures, allowing the identification of conserved and unique fragments through comparisons of genome sequences both with other species and among themselves.

Comparative analysis of the sequenced Msi and sorghum genomes (Mitros et al., 2020) revealed that each sorghum chromosome corresponds to a pair of Msi chromosomes, with the exception of a single Miscanthus chromosome. Specifically, this chromosome corresponds to the fusion of two sorghum chromosomes. Analysis of the Msi genome revealed synteny between Miscanthus and S. bicolor genome sequences. The Msi DNA appears to contain DNA similar to that of the ancestor of modern sorghum, the amount of which was doubled during chromosome fusion events; consequently, the genus Miscanthus comprises paleoallopolyploids (Ma et al., 2012; Mitros et al., 2020; Trieu et al., 2022).

Thus, during their evolution prior to diversification, but after diverging from the closely related sugarcane clade, species of the genus Miscanthus underwent a stage of ancestral genome tetraploidization and subsequent chromosome fusion (Swaminathan et al., 2012). As a result of diploidization, Miscanthus spp. possess two homeologous chromosomes that are highly syntenic, potentially providing functional redundancy between homeologous gene pairs (Trieu et al., 2022).

## Studying the structure of target genes based on whole-genome assemblies

Whole-genome assembly enables complete and highly accurate identification of the set of protein-coding genes in Miscanthus, including genes that are targets for breeding based on the most important biotechnological traits.In the study dedicated to the whole-genome assembly of Msi (Mitros et al., 2020), an analysis of seasonal dynamics of gene expression in leaves, stems, and rhizomes of plants was conducted. The authors showed that variations in gene expression levels between different organs exceed seasonal variations. Nevertheless, for genes within a single tissue, seasonal changes significantly influenced expression levels. Expression data allowed the identification of several functional gene clusters related to important functions: nitrogen metabolism; amino acid metabolism; hormonal regulation and signaling; genes associated with pathogen response; and genes involved in starch and sucrose biosynthesis. Among these gene groups, key genes were highlighted based on expression data. A network of seasonal nutrient uptake and utilization was reconstructed.

In the study on the Msa genome assembly (De Vega et al., 2021), reconstructed sequences of protein-coding genes enabled the search for orthologs in Msi, as well as in other plant species (foxtail millet Setaria italica, sorghum S. bicolor, maize Z. mays, switchgrass P. virgatum).

In the study on the Mfl genome reconstruction (Zhang et al., 2021), considerable attention was devoted to investigating the structure of cellulose biosynthesis genes – cellulose synthases. Within this group of enzymes, based on ortholog searches considering synteny of genomes of both Miscanthus and rice, sorghum, and sugarcane, several functional clusters were identified, whose origin is associated with duplication and expansion events of this family in Miscanthus.

In the study on the Mlu genome assembly (Miao et al., 2021), the focus of protein-coding gene research was on functional annotation analysis and patterns of expansion and contraction of gene families. The proportion of gene families subject to expansion exceeded the number of families with gene losses by nearly threefold (9,509 versus 3,228). A total of 211 gene families demonstrated rapid expansion in Mlu; these included pathogen resistance genes (containing the NB-ARC domain), genes associated with xylanase inhibition function, salt stress response genes, cytochromes P450, terpene synthases, and several others.

A separate analysis was dedicated to genes of the C4 photosynthesis pathway. These were identified based on orthology with sorghum genes, totaling 55. Most of these genes in Mlu underwent expansion through proximal, tandem, and segmental genomic duplications. As a result, their number was almost twice that found in sorghum.

## Conclusion

The high biotechnological potential of perennial grasses of the genus Miscanthus for human economic activities, their broad adaptive capabilities, and their ability to maintain high biomass productivity under unfavorable abiotic and biotic stress conditions – all of these factors make them a valuable target for breeding and industrial applications. The adaptive potential of Miscanthus species (Msi, Msa, Mlu, and Mfl) is underpinned by conserved and species-specific features of genome organization. At the same time, the hybrid M × g, derived from crossing Msa and Msi, is characterized by seed sterility and extremely low genetic diversity, rendering it vulnerable to diseases. To overcome this limitation in Miscanthus breeding, working with diverse hybrids can be recommended.

Modern technologies for whole-genome genotyping, sequencing, genome assembly, and bioinformatics provide opportunities for in-depth investigation of genetic diversity within the genus Miscanthus, as well as genome structure and gene repertoire. This enables the identification of genetic control mechanisms underlying traits that are key to the industrial potential of this crop. The complete gene set of Miscanthus allows reconstruction of gene networks and metabolic pathways, and study of the impact of genetic variation on the functioning of key molecular and physiological processes in the plant. Such research, utilizing an expanded set of sequenced genomes, will form the foundation for targeted Miscanthus breeding programs.

## Conflict of interest

The authors declare no conflict of interest.
